# Biobanks and data interoperability in Latin America: engendering high-quality evidence for the global research ecosystem

**DOI:** 10.3389/fmed.2024.1481891

**Published:** 2024-12-16

**Authors:** Erick Valdés, Juan Alberto Lecaros

**Affiliations:** Institute of Sciences and Innovation in Medicine, Facultad de Medicina Clínica Alemana Universidad del Desarrollo, Santiago, Chile

**Keywords:** biobanks, regulation, data interoperability, high-quality evidence, LATAM biobanks network, reproducibility of scientific studies

## Abstract

Currently, each biobank in Latin America operates with its own set of standards for database creation and management, resulting in a lack of regional and international interoperability. Furthermore, regulations concerning data protection, curation, and the transfer of biological samples and associated data vary significantly from country to country, by complicating efforts to create a unified data-sharing platform. To address these challenges, Latin America should promote the development of an integrated regional network of biobanks to generate high-quality evidence within the global research ecosystem. This initiative will combine regulatory science—focused on interoperability standards across semantic, technical, legal, and organizational dimensions—and meta-science, which assesses the quality of scientific practice. Evidence indicates that harmonized standards in biobanks lead to higher-quality, more reliable data, thereby facilitating the reproducibility of scientific studies. This paper aims to identify and address existing regulatory, policy, and infrastructure gaps in Latin America to establish harmonized interoperability criteria essential for reproducing biomedical studies. Additionally, it seeks to propose minimum standards for regulating biobank networks, which will promote the development of medical products on a global scale, thereby engendering high quality evidence for the global research ecosystem and enhancing Latin America’s integration into it.

## 1 Introduction

Biobanks are not simply collections of human biospecimens; they are organizations that must adhere to ethical and legal standards within a structured governance framework. These entities, whether part of public or private institutions, are dedicated to biobanking—the process of acquiring, storing, and managing defined biological materials, along with all associated data and information. This includes a range of activities linked to collect, prepare, preserve, test, analyze and distribute such biological samples and associated data (1). Therefore, biobanking implies working with materials that contain personal information, such as health records, family backgrounds, lifestyle, and genetic information, among others (2).

It is important to distinguish between biorepositories and biobanks, as the former lack the governance principles that enable the regulated transfer of samples to researchers. In contrast, biobanks are devoted to store and manage human material in order to obtain clinical outcomes and, as such, they need specific governance mechanisms to allow third parties to access to those resources in a systematic, efficient and regulated way (3). However, although biorepositories are originally intended for specific research and are under the responsibility of a researcher, in the long-term they can be important parts in the constitution of biobanks, insofar as these collections are of high quality and traceable.

One of the primary goals of biobanks is to provide high-quality biological materials and data for medical research, ensuring the reproducibility of studies (4). The role of biobanks in enhancing reproducibility is becoming increasingly important to (i) support regulatory decisions, (ii) represent and interpret data for analyzing regulatory successes and failures, and (iii) contribute to the integration of regulatory science and meta-science approaches, which can significantly improve the quality of regulated medical products and practices.

Over the past few decades, biobanking has been a key driver of biomedical research—particularly in precision medicine—by enabling the identification of biomarkers and potential treatments based on genomics and phenotyping (5). In this context, data interoperability is crucial for harmonizing the collection standards for biological samples and associated donor data, such as social, environmental, or lifestyle information. Enhanced data interoperability allows for the generation of clinical data that can be compared across datasets from various geographical regions, facilitating the combination of information from different sources and biobanks, and thereby increasing the number of patients and data involved.

Latin America (LATAM) has yet to establish a cohesive international biobank infrastructure. Each country has developed its own standards for the creation and management of biospecimens and databases, resulting in a lack of regional and international interoperability. Furthermore, regulations concerning data protection, data curation, and agreements on the transfer of biological samples and data differ significantly across countries, creating challenges in forming a unified platform for data sharing (6–8).

Biobanks in LATAM could play a key role in generating high-quality evidence within the global research ecosystem by integrating regulatory science—covering interoperability standards at semantic, technical, legal, and organizational levels—and meta-science, which evaluates the quality of scientific practices (9). However, regulatory, policy, and infrastructure gaps currently hinder LATAM countries from establishing a coordinated biobanking ecosystem. This paper aims to address these gaps by proposing harmonized interoperability criteria, essential for the reproducibility of biomedical studies. Furthermore, it advocates for the creation of a LATAM Biobanks Network to establish a coordinated biobanking ecosystem and recommends minimum standards for the regulation of biobank networks and medical products at a global level.

## 2 Assessment of biobanks’ policy in LATAM

Latin America has various collections of biological samples used in non-standardized research, as well as a few biobanks that manage both public and private collections. However, the absence of national policies governing their establishment and operation has resulted in lack of regulation in several countries (except Colombia and Brazil), fragmented efforts, and limited funding (6, 10, 11). This situation hampers the region’s capacity to produce impactful biomedical research at both national and international levels, perpetuating the underrepresentation of Latin American populations in genetic databases and global collaborative research (12). The insufficient funding for most biobanks jeopardizes their operational sustainability, staff training, and ability to engage in projects of national and international significance. Moreover, the absence of strategic population biobanks limits the state’s capacity to address urgent research needs and develop public policies in response to emerging infectious diseases.

### 2.1 Biobanks regulation in LATAM

Although the literature on biobanks in Latin America remains limited, these institutions have gained significant prominence in national policies over the past few decades. For instance, in Colombia, Law 2287/2023 was enacted last year, establishing the National Biobank System and regulating biobank operations for biomedical, biotechnological, and epidemiological research, among other provisions. Similarly, in Argentina, Resolution 336/2023 was passed to create the National Biobank of Biological Samples Associated with High-Impact and/or Relevant Pathologies. In Brazil, the Resolution 441/2011 of Health National Council was enacted to fill the legal vacuum that had existed until then regarding the storage and use of human biological material for research purposes. In countries lacking specific biobank legislation, such institutions operate under personal data protection and clinical trials regulations, as is the case in Chile (13, 14).

If effectively interconnected and enhanced, biobanks could play a pivotal role in LATAM, particularly in advancing biomedical research, elucidating disease mechanisms, and fostering personalized medicine. As precision preventive medicine evolves, biobanks will be instrumental in shaping healthcare strategies and personalized public health by integrating genomic, transcriptomic, proteomic, metabolomic, and epigenomic data with population health information. LATAM biobanks could make significant contributions to the discovery of new biomarkers for colon, pancreatic, and thyroid cancers (15). Despite their current limitations, biobanks have already played a crucial role in generating genetic profiles for KIT gene mutations, which assist in predicting drug sensitivity—such as for Imatinib—and optimizing chemotherapy by preventing adverse reactions and ensuring appropriate dosing for patients (15).

A recent study identified 44 biobanks across LATAM, with the majority (89%) focused on specific diseases, while the remaining 11% are population-based. According to this study, Chile counts on 11 biobanks,^1^ followed by Mexico with 10 and Argentina with 6. In most countries, regulatory frameworks for biobanks are either weak or non-existent, with Brazil having the most developed structure (6). However, this study disregards the official data presented by Comissão Nacional de Ética em Pesquisa (CONEP), the highest instance of ethical evaluation in research protocols involving human beings in Brazil, which in July 2024 published the general map of 103 approved biobanks.^2^ Most biobanks in LATAM are primarily focused on COVID-19, recruiting and storing biological samples for research and education. They also collect non-neoplastic tumor samples and tissues from various anatomical regions, preserve genetic material related to oncohematological diseases, and study bile duct and biliary cancers. Additionally, they contribute to advancing cancer epidemiology, by conducting observational studies on the effects of cancer interventions and environmental disruptors, researching Alzheimer’s and other neurodegenerative diseases, and developing new antimicrobials, among other areas (6).

Due to the independent operation of these biobanks and the lack of a regulatory framework to facilitate systematic collaboration, their interoperability and governance, as well as the data they collect and store, are severely limited. This absence of networks and alliances hinders the region’s ability to efficiently share data and metadata associated with the biological samples housed in these institutions. In contrast, Europe is advancing data management initiatives through research projects such as BioSHaRE-EU, and through the implementation of standardization policies, including both soft law and ISO standards (16). Biobanks in Latin America should work as interoperable, structured research platforms designed to meet the needs of participants, society, and the scientific community. Their success fundamentally depends on building trust with the public and key stakeholders. However, trust cannot be secured without robust mechanisms that ensure not only interoperability and governance but also transparency, ethical practices, equity, quality, and the relevance of biobank activities as stewards of the samples and data entrusted to them. Once such a framework is established, LATAM biobanks will need to implement and uphold processes that guarantee the quality and usability of samples and data while safeguarding participants from risks. This will be achieved through adherence to shared, standardized practices.

Thus, biobanks in LATAM will be established as institutional benchmarks, demonstrating a strong commitment to the common good through public accountability and transparence. They will ensure the quality, traceability, ethical reuse, and secure handling of samples and data. This will be accomplished through ongoing, mutually beneficial relationships with participants, stakeholders, and society at large. In this context, LATAM biobanks and potential biobank networks must establish governance, regulations, and operational procedures that ensure these objectives, characterized by standardized processes and well-organized resources.

However, there are several gaps that LATAM biobanks must address, particularly regarding regulatory, policy, and infrastructure issues (6, 10, 11). From a regulatory perspective, the region lacks specific regulations to ensure the legal security of biobank operations and adequately protect the rights of individuals donating biological samples for research purposes. The existing regulatory frameworks, which generally pertain to biomedical research, are clearly insufficient to govern this specialized activity.

Despite the efforts of various government and industry stakeholders, an adequate regulatory framework for research and innovation in the biomedical field remains absent. Companies frequently report that regulations for specific technologies are either nonexistent or insufficiently defined. Additionally, when regulatory agencies are in place, they often lack trained personnel with expertise in biobank activities, resulting in delays in the approval process.

The absence of regulations with a broader and more comprehensive approach hinders the protection of donor rights, including autonomy, confidentiality, privacy, and access to data and research results. Furthermore, the lack of clear guidelines regarding the import and export of samples, intellectual property, and the overall legal security of biobank operations presents significant challenges. These regulatory gaps impede the establishment and effective functioning of biobank networks in the region.

Modern legislation for the protection of personal data and a robust regulatory framework for genomic and clinical databases stored in public repositories remain inadequate. For instance, in Chile, Law 19.628 on personal data does not sufficiently safeguard sensitive information, such as health and genomic data. Since 2017, a bill (Draft Law on the Protection of Personal Data, bulletins N°11.092–07 and N°11.144–07) has been under discussion to amend Law 19.628. This proposed legislation aims to regulate data protection and processing while establishing a Data Protection Agency, with the goal of aligning Chilean law with OECD standards and adhering to GDPR guidelines. Similar issues are observed in other countries throughout the region.

Regarding policy, there are few government initiatives that promote the establishment of cohorts to collect biological samples and associated data on prevalent diseases in the general population or specific ethnic groups, in accordance with international standards of quality and interoperability for biomedical research (13, 14, 17–21). This lack of support significantly hampers research progress and the development of products and strategies applicable to clinical settings, thereby obstructing the advancement of public health policies for the population.

Notwithstanding some efforts by government and private sector actors, most countries in LATAM lack effective coordination policies between translational medicine research and local pharmaceutical and biotechnological industries. Furthermore, recent initiatives by government agencies to harmonize and standardize data and sample management have not been successful. As a result, persistent deficiencies continue to hinder interoperability and limit opportunities for both national and international collaborations. While Chile, Brazil, Argentina, and Colombia show significant scientific capacity, there are currently no public policies that ensure access to collections meeting international standards (6). Such standards are essential for guaranteeing the quality of samples and associated data for both current and future high-quality research.

There is a noticeable absence of effective strategies and policies to ensure the return of remaining biospecimens and research findings obtained through international collaborations. The scientific community in Latin America lacks a strong biobanking culture and faces a shortage of advanced human capital specialized in biobanking. Additionally, local Ethics Review Committees often lack the expertise and knowledge needed to evaluate projects involving broad consent for the use and storage of human biological samples in biobanks (22).

With respect to material resources, biotechnological companies encounter significant challenges in accessing infrastructure and specialized services from certified laboratories or centers for preclinical studies, toxicology, biobanks, and other essential areas (23). Consequently, these companies are often compelled to seek solutions abroad, leading to additional costs, or to rely on services and products of suboptimal quality.

The absence of a coordinated international biobank network dedicated to promoting high-quality scientific research leads to wasted resources and diminished returns on investment. This inefficiency arises from the repeated establishment of similar cohorts without adherence to quality standards or proper preservation of biological samples and data.

In summary, LATAM faces a diverse regulatory landscape concerning legislation on the reuse of samples and data. This presents several challenges in the region, including:

- Developing specific regulations for biobanks and sample collections, as existing laws and policies are inadequate in ensuring donor rights and defining stakeholder obligations.
- Establishing connections between regulatory authorities and issues related to biobank regulation.
- Training Ethics Review Committees (ERCs) to support biobank governance and prevent operations without proper oversight.
- Harmonizing data protection legislation across the region.
- Reaching consensus on soft law or other regulatory measures to facilitate interoperability and governance.

These challenges also introduce methodological issues that impact data interoperability among biobanks in LATAM.

### 2.2 Methodological issues when engendering data interoperability of biobanks in LATAM

Data and metadata associated with biological samples are essential for clinical and biomedical research. Consequently, access to and availability of these resources through a biobank must be meticulously structured. Proper data validation panels are critical, as they ensure scientific reproducibility by clarifying the characteristics of data acquisition and processing for researchers (24).

In Europe, for example, the Minimum Information About Biobank Data Sharing (MIABIS) standard aims to standardize the data elements used to describe biobanks, research on samples, and associated data (25). This standard helps create a common language among biobanks for sharing data and metadata related to available samples (26, 27). However, a similar tool, policy, or standard is not yet available in LATAM.

The following sections will outline the standards that should be considered when proposing an interoperability policy for the region.

#### 2.2.1 FAIR vs. open data

Biobank data can adhere to standards such as Open Data or FAIR within the open research ecosystem. The FAIR methodology emphasizes data that is Findable, Accessible, Interoperable, and Reusable. This approach has been adopted by the European Union and became a mandatory standard for EU-funded scientific research in 2020 (28, 29).

In the FAIR methodology, accessibility is governed by rights and ethical principles designed to protect the privacy of sensitive data, ensuring that it is “as open as possible and as closed as necessary” (30). In this context, the General Data Protection Regulation (GDPR) plays a crucial role in Europe, regulating health data in accordance with FAIR principles.

The central idea is that while Open Research emphasizes data access and reuse to inform public policies and decisions in health and science, such data must be used or reused responsibly (30). In contrast, the Open Data methodology defines data as being available for use by the public without restrictions, although private, confidential, or classified data is generally excluded (31). The goal is for this data to be interoperable and freely reusable (30). This policy does not specify principles for authentication or access but instead focuses on minimizing the cost for users who wish to reuse the data. Additionally, it does not address the purpose for which the published data will be used or reused (30).

The reuse of clinical data and the absence of centralized privacy standards highlight the necessity of the FAIR methodology, especially in light of the limited and outdated legislation concerning sensitive and clinical data in LATAM.

According to recent studies (9, 32, 33), we can argue that the FAIR methodology is critical for data management and biobanking for several reasons. First, it improves data discoverability (Findable). By adhering to FAIR principles, data become easily identifiable through standardized metadata and unique identifiers. This is particularly important for biobanks, as researchers must efficiently locate relevant biological samples and associated data for their studies. For instance, biobanks can use standardized metadata to describe samples, making it easier for researchers to find specific datasets or biospecimens relevant to their research interests.

Second, it improves data accessibility (Accessible). FAIR encourages the development of clear access protocols, ensuring that data can be accessed by authorized users without unnecessary barriers. This is especially important for collaborative research and international studies. For example, implementing secure data access methods allows researchers to use data from biobanks while protecting participant confidentiality and ensuring compliance with ethical standards.

Third, it promotes interoperability (Interoperable). By adhering to common standards and protocols, data from different biobanks can be integrated and analyzed together. This is essential for large-scale studies that require diverse datasets from multiple sources. As an example, we can see that using standardized formats and vocabularies enables biobanks to share data with other institutions and platforms, facilitating broader research collaborations and improving the overall quality of scientific inquiry.

Fourth, it ensures data reusability (Reusable). FAIR principles promote the use of clear licenses and terms of use, allowing data to be reused in future research projects. This maximizes the value of the resources invested in data collection and management. For instance, providing detailed documentation about how data were collected, processed, and can be reused helps ensure that future researchers can confidently utilize biobank data in their studies.

Fifth, it supports regulatory compliance. The FAIR methodology aligns with many regulatory and ethical guidelines in biobanking, which require transparency and accountability in data management. This is crucial for maintaining public trust and ensuring compliance with local and international regulations.

Sixth, it facilitates innovation. By promoting data sharing and collaboration, the FAIR framework fosters innovation in biomedical research. Researchers can build upon existing data, leading to new insights and advancements in fields such as personalized medicine, genomics, and public health.

Seventh, it enhances scientific collaboration. FAIR principles facilitate collaboration among researchers, institutions, and countries by creating a common understanding and framework for data sharing. This is particularly important in fields like epidemiology and public health, where comprehensive datasets are essential for understanding complex health issues (9).

In summary, the FAIR methodology is vital for biobanking and data management as it enhances discoverability, accessibility, interoperability, and reusability of data (9). By implementing these principles, biobanks can significantly improve research outcomes, foster collaboration, and contribute to the advancement of scientific knowledge while ensuring ethical standards and compliance with regulations.

#### 2.2.2 Interoperability and harmonization standards

The FAIR methodology underscores the importance of data harmonization to ensure interoperability and facilitate sharing. Effective harmonization requires a common, interoperable language for the data stored by biobanks. In contrast, Open Data policies do not establish these parameters in advance.

Data harmonization involves converting disparate and inconsistent data from various sources into a unified format. Without this process, managing large datasets or “big data” becomes increasingly challenging. Several ethical considerations are crucial in the management of digital data, particularly given that ethical standards vary by country. Key issues include data sharing and ownership, informed consent, international collaboration, transfer of biospecimens, returning research results to participants, benefit sharing, and the role of ethics committees (34). Developing consensus guidelines is essential for improving the ethical standards associated with data harmonization.

Common Data Elements (CDEs) and standardized vocabularies are crucial for enabling researchers across different regions to utilize and cross-reference data with other databases, resulting in more accurate findings. This is especially important when combining samples from multiple biobanks, particularly in diseases with low patient recruitment numbers in each region. The need for such standardization is partially addressed by the ISO/IEC 11179 standard, which encompasses common vocabularies for data and metadata descriptions (35). In the context of the harmonization standards, it is important to take into account the latest edition of the *ISBER Best Practices: A New Process for Relevance in an Evolving Landscape*,^3^ as a relevant global guide for managing and operating biobanks (specifically Section I: Information Management).

In LATAM, the adoption of the international ISO standard for CDEs and the establishment of a common language for potential biobank networks remain unresolved challenges.

#### 2.2.3 Data quality

ISO/TR 22758:2020 provides guidance for the implementation of ISO 20387 and is essential for ensuring consistent minimum quality standards for samples and associated data in biobanks. Similarly, ISO/TC 276 on biotechnology establishes key parameters for annotating and standardizing data to maintain its overall quality. The FAIR methodology offers a measurable set of criteria for defining high-quality data (36), and its adoption would significantly enhance data quality practices in research.

Matera-Witkiewicz et al. (35) emphasize that, despite the availability of advanced technological tools for big data analysis, meaningful, actionable, or accurate clinical results cannot be achieved if the data is of poor or insufficient quality. Similarly, Lu et al. (37) argue that the critical factor in clinical or biological analyses is not the volume of data, but rather ensuring that essential data and metadata from samples are accessible and meet quality standards for reproducibility.

Given the diverse regulatory landscape for biobanks in LATAM, developing proposals for policy harmonization and governance is crucial. Standardizing criteria for data protection, achieving multilateral consensus on data interpretation and representation, and establishing unified guidelines are critical steps. These measures will facilitate the generation of high-quality evidence, benefiting both regional and global research ecosystems.

Furthermore, metadata is essential for biobank collections, particularly when sample materials are not directly accessible to researchers (24). Biobanking intersects with various fields, including medicine, biology, systems biology, information technology (IT), artificial intelligence (AI), machine learning, modeling, mathematics, statistics, and big data (38). As a result, effective data management and interoperability are crucial for advancing multiple research domains.

Therefore, it is advisable for countries to align with ISO/TC 276 on biotechnology and its associated data standards, as well as the updated ISO 20387, to ensure the effective operation of biobanks at both national and international levels.

## 3 Policy actionable recommendations

Our region faces several significant challenges regarding its biobank infrastructure that must be addressed to generate high-quality research evidence and establish global standards for medical product regulation. We think that the most critical deficiencies include:

1. ***Disjointed Management***: Ineffective coordination in managing stored samples and the data derived from them.
2. ***Inconsistent Legal Frameworks***: Varied legal interpretations of the role, nature, and purpose of biobanks across different jurisdictions.
3. ***Lack of Usage Guidelines***: Insufficient information on how samples and data should be used, who can use them, and for what purposes.
4. ***Absence of Models and Structures***: No established frameworks or decision-making models for the reuse of samples and data.
5. ***Coordination and Capacity Issues***: Limited ability to develop methods ensuring the quality of FAIR metadata, which is crucial for the effective reuse of samples and data.
6. ***Funding Shortages***: Inadequate financial resources to build and maintain biobanks and data collections.
7. ***Weak Regional Cooperation***: Insufficient interjurisdictional collaboration at the regional level.
8. ***Lack of Legislative Will***: Limited local legislative commitment to addressing these issues.

Some of these issues are influenced by political will, which is beyond our control. Therefore, we will concentrate on technical aspects that can be optimized across regions. With this focus, we present three proposals aimed at enhancing biobank regulation and governance, establishing consistent standards, generating high-quality evidence, and integrating our region into the global research ecosystem.

### 3.1 Advancing toward an articulated interpretation of shared ethical and legal concerns

The current legal systems in LATAM are fragmented concerning a common objective: the interoperability and governance of biobanks in the region. We propose the establishment of a policy working group comprising biobank experts and professionals with expertise in technical, ethical, and legal matters. This group would be tasked with developing legislative and public policy proposals to promote regulatory harmonization and recommend common standards for biobank governance. Such an initiative could improve mechanisms for sharing samples and data within LATAM through harmonized policies and bilateral or multilateral agreements. Additionally, it would establish transparent and standardized governance and interoperability criteria for biobanks across the region.

### 3.2 Training and investment to improve data quality and engender opportunities for sample reuse

Overall, biobanks in LATAM are significantly underfunded and often lack public financial support. Scientists and researchers frequently encounter financial constraints that hinder their ability to maintain samples and conduct long-term longitudinal studies. This lack of funding raises concerns about the capacity of biobanks to deliver reliable research with legal security. Consequently, both the private and public sectors are hesitant to invest in new infrastructure or the reuse of samples and data, which adversely affects the quality of the samples and their future utility. This situation creates a vicious cycle that not only compromises the technical quality of biobanks but also impedes their interoperability and full integration within the research ecosystem.

We propose collaborating with the private sector to explore new funding opportunities and innovative funding models for physical biobanks, sample collections, and related institutions. While funders and sponsors may have competing priorities and limited capacity for engagement, forming a coalition of funders can help mitigate risks and develop a unified funding agenda. This strategy can accelerate progress and enhance the precision of public health responses by ensuring access to high-quality, comprehensive data and samples. LATAM should pursue a vision of precision public health by: (i) building public and private support for biobanks and sample collections; (ii) collecting FAIR metadata to describe samples, thereby facilitating informed investment and reuse; (iii) offering online and in-person workshops to support capacity building, with a focus on data and sample quality as well as ethical sample reuse; and (iv) fostering a network of “biobankers” to shift attitudes and promote biobanks.

### 3.3 Establishing a LATAM biobanks network (LABN)

We have identified key gaps in the region that hinder the establishment of advanced biobanks capable of creating strategic public collections with harmonized legal and technical regulations in accordance with international standards. Therefore, we emphasize the importance and urgency of developing a LABN to address both national and international needs, whether public or private. This network would foster innovation in health technologies and policies by serving as a public good. It would provide top-tier scientific, technical, and technological support to projects, offer training in ethical, regulatory, and best practices for biobanks, and enhance the societal value of these institutions. By enabling the coordinated and standardized creation of population collections on both national and international scales, the network would capture the genetic diversity of the region’s countries. This would equip LATAM to respond efficiently and rapidly to research needs, including potential future pandemics.

Operationally, the network will leverage existing experiences and capabilities within the countries, enhancing them through a hybrid governance model that combines the advantages of a federated system with centralized coordination. Each country with biobanks should establish at least one high-complexity biobank, which will serve as a conduit between the regional biobanks and an International Coordination Platform. This platform will be responsible for developing operational policies for the network, ensuring quality and securing foundational public funding for the nodes, and maintaining comprehensive records of collections and data stored within the biobank network.

**Figure 1:**
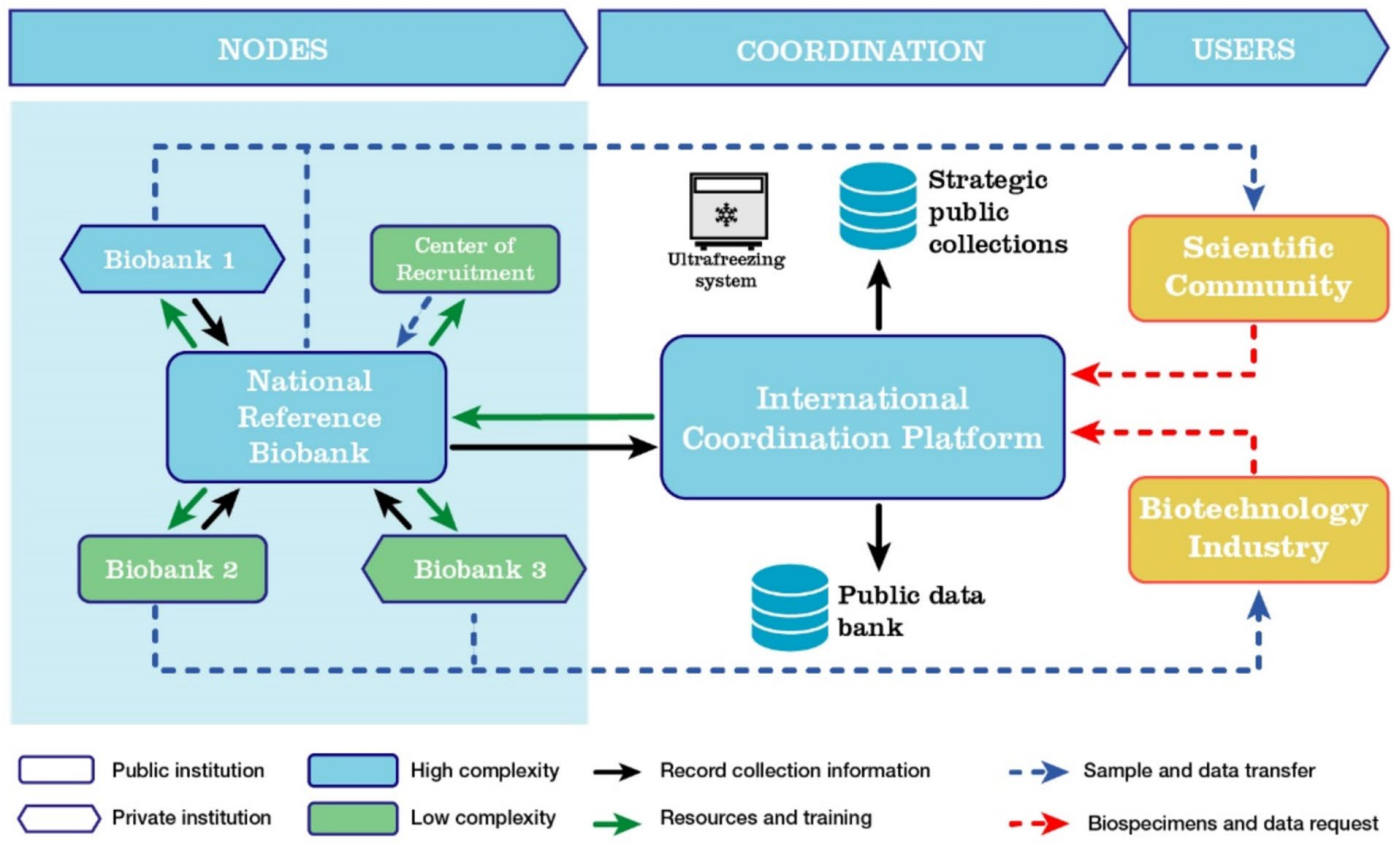
Organization of the LATAM biobanks network as a mixed network. In each country, a high-complexity biobank will collaborate with the International Coordination Platform to oversee and coordinate the network. Lower-complexity biobanks will be connected to and trained by the reference biobanks, while maintaining their own autonomous operations.

The network will include biobanks from both public and private institutions, each offering varying levels of access to their collections. These biobanks will register samples and associated data to facilitate transfer requests from interested parties, including both public and private sector. Additionally, the platform will coordinate public collections of strategic interest, enabling participating network nodes to actively engage in these initiatives (Figure 1).

The structure of a biobank network promotes integrated collaboration across clinical, scientific, and biotechnological fields, creating a synergy that accelerates biomedical research and the development of new health technologies. Biobank networks are emerging as a form of Big Science, supported by extensive cryopreserved sample collections, comprehensive clinical and epidemiological databases, genomic and proteomic pipelines, and advanced computational and storage resources for data analysis (39). Consequently, biobanks are evolving from mere infrastructure into a critical component of medicine, public health, and biomedical biotechnological advancement.

Some of the advantages offered by biobanks networks include:

(a) *Research:*

- Greater quantity and quality of samples and data.
- Technical, legal, and ethical advice.
- Standardization of processing.
- New basic-clinical collaborations.
- New sources of funding.
- Resource optimization.
- Supports multicentric and clinical studies.

(b) *Industry:*

- Reference center provider.
- Prototype evaluation.
- Cost savings.
- Faster development of diagnostic, prognostic techniques, and drugs.

(c) *Patients:*

- Earlier and more cost-effective diagnosis.
- Prognostic systems.
- New therapeutic targets.
- Personalized medicine.

(d) *Population:*

- Identification of risk factors.
- Diagnostic tool for prevention.
- Improvement of public health policies.
- Enhanced health equity.

**Figure 2:**
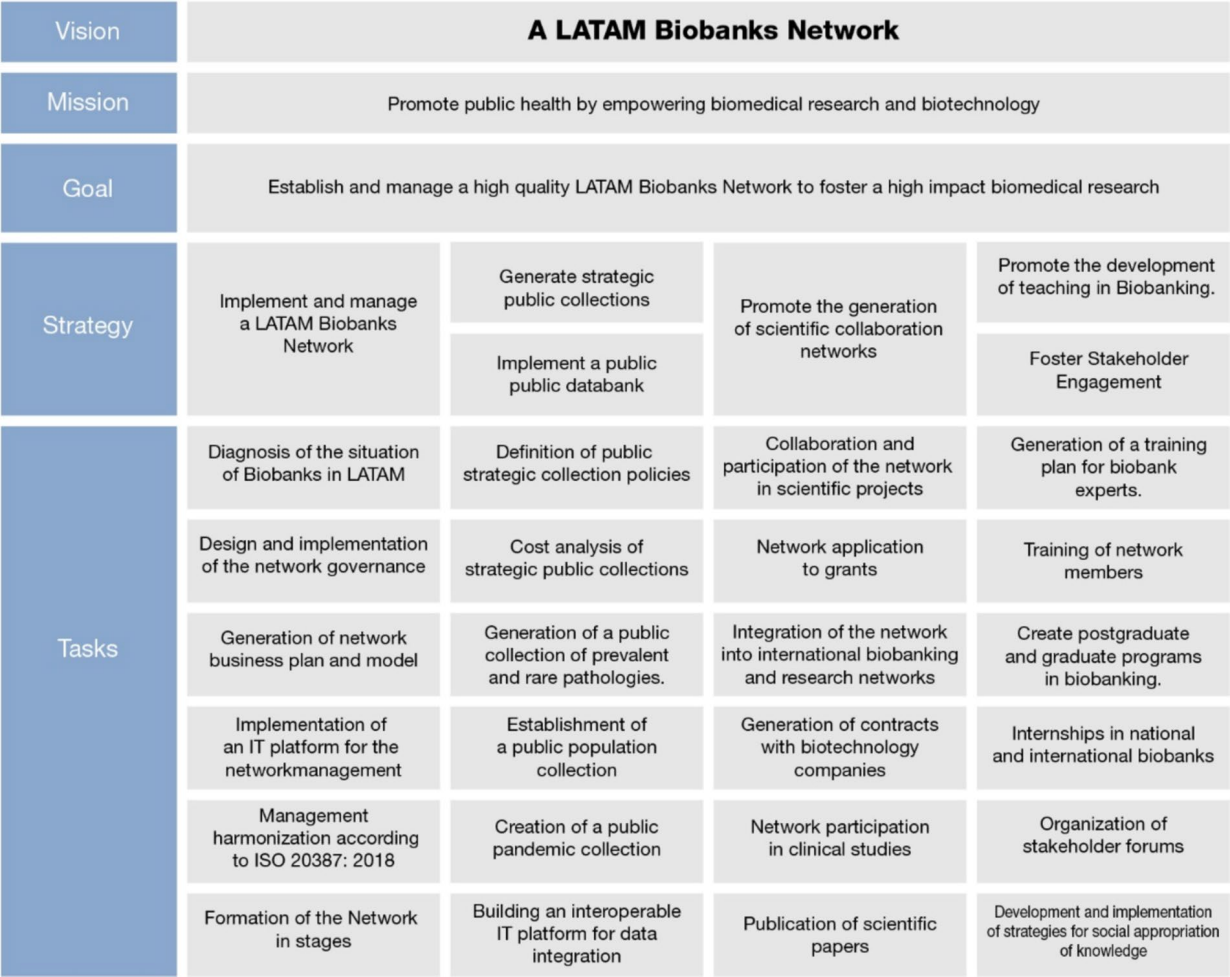
Description of the strategic plan of the LABN.

In summary, we propose the establishment of a LABN comprising various disease-focused and population-based biobanks. These biobanks would operate in a coordinated and harmonized manner to provide the scientific community with high-value and high-quality biological resources and associated data from the LATAM population. This network would enhance the development of both national and international biomedical and clinical research, foster innovation in health technologies, and contribute to the improvement of health policies in our countries. This LABN should be established by following both a development plan and a strategic framework, which can be schematized in Figure 2.

## 4 Comparative standards for the regulation of biobanks networks and medical products at a global level

Research biobanks’ activities must adhere to ethical standards recognized in international guidelines, recommendations, or declarations on human research ethics, such as the *International Ethical Guidelines for Health-Related Research Involving Humans* (40). Additionally, biobanks are regulated by legally binding norms established by national laws or supranational and international legal instruments that protect research subjects in biomedicine and safeguard their personal health or genetic data.

The increasing complexity and internationalization of biobank operations have expanded the regulatory frameworks governing this activity beyond the initial recognition of basic donor rights, such as autonomy, informational self-determination, privacy, and confidentiality of personal data (41, 42). Current biobanks regulations now also encompass donors’ rights to be informed of the overall results of research conducted with their samples, to access their personal data, and to choose whether to receive or decline relevant health information (the right to know and not know) (43–45). Additionally, they address the availability of genetic counseling when applicable, along with other ethical and legal aspects related to governance, such as international data sharing, and the biobank’s relationship with the community (46).

The rapid growth of biobanks in Europe and the United States, particularly since the early 2000s, has led legal systems to respond by enacting specific regulations for biobanks. These regulations are primarily aligned with existing laws on scientific research involving human subjects and personal data protection, or they apply these general laws to the activities of biobanks (47, 48).

The strategy of implementing specific legal regulations for biobanks was adopted early on by several European countries, which initiated national biobank policies (49). Iceland (Act on Biobanks, 2000), Estonia (Human Genes Research Act, 2000), and Sweden (Biobanks [Health Care] Act, 2003) were among the first to enact specialized laws to regulate the operation of population-based biobanks, including clear provisions for broad informed consent. In contrast, other countries with national (United States and United Kingdom) or federated (Canada, Denmark, Netherlands) biobanks did not establish special laws for this purpose but instead relied on guidelines and general legislation applicable to biomedical research and personal data protection (50, 51).

In the United Kingdom, following the establishment of the UK Biobank in 2002, the Human Tissue Act (2004) was enacted. This legislation established the Human Tissue Authority, which is responsible for licensing biobanks and overseeing the collection, storage, and use of human tissues. In the United States, there is no federal law specifically regulating biobanks. Instead, biobank activities are governed by a range of general laws, including the Common Rule (45\u00B0C.F.R. § 46), the Health Insurance Portability and Accountability Act (1996), the Standards for Privacy of Individually Identifiable Health Information (commonly known as the ‘Privacy Rule’), and the Privacy Act (1974). Additionally, laws such as the Stem Cell Therapeutic and Research Act (2005) and the Genetic Information Nondiscrimination Act (2008) are relevant to biobanking activities. However, the absence of dedicated biobank legislation in the United States has been criticized for complicating the application of general rules to biobanking practices and for inadequately protecting personal data associated with samples, particularly in terms of de-identification as required by European standards (48).

Comparative legal analysis reveals a general trend toward allowing the use of broad consent. This involves obtaining a single consent from donors, while each research study utilizing the samples and associated data must still receive prior approval from an ethics committee. This approach aligns with the purpose of biobanks, which is to enable samples to be used for a range of future studies. This trend is evident in both countries with specific legislation and those relying on general norms and specific guidelines. It is important to distinguish this type of consent from a blanket one, which involves a single consent for all future research without requiring separate ethics committee approval for each study—a practice widely regarded as ethically and legally problematic.

Other key aspects of biobank regulation identified through comparative law include data security and privacy measures, such as coding, anonymization, or de-identification, as well as the authorization or prohibition of international data exchange. A prevailing trend is to allow international data sharing, provided that the recipient country maintains an adequacy level of data protection with respect to the country from which the samples originate (48).

Within the framework of the international human rights system, the UNESCO International Declaration on Human Genetic Data (2003), although non-binding, is one of the first international instruments to establish specific guidelines for the collection, processing, use, and preservation of human biological samples for research purposes—whether scientific, judicial, or forensic—as well as for the personal data that may be derived from them. This declaration complements the UNESCO Universal Declaration on the Human Genome and Human Rights (1997), which sets out legal principles concerning the protection of the human genome in diagnostic, therapeutic, and research contexts.

In the European context, biobank activities are regulated by the Council of Europe’s Recommendation CM/Rec 619 (2016) on the use of human biological samples for biomedical research. Chapter IV (Articles 15 to 20) of this document outlines governance standards for what it refers to as ‘collections.’ It establishes basic governance principles, including the designation of a responsible party, the specification of purpose, procedures for changing the purpose, quality assurance measures, security and confidentiality protocols, closure procedures, management and use information, and requirements for annual activity reports. The recommendation also mandates feedback to donors about health-relevant findings, transparency mechanisms for sample access, policies for cross-border sample flows, and oversight mechanisms.

The development of harmonized regulations at the European level, as previously highlighted, has facilitated the creation of robust biobank networks across the continent. A notable example is the Biomolecular Resources Research Infrastructure (BBMRI), launched in 2013, which aims to develop an advanced information technology framework for data exchange between biobanks and implement strategies to ensure the quality management of biological materials. In contrast, the lack of a similar framework in LATAM hinders local initiatives from integrating into the collaborative networks established by European countries.

In light of the points outlined above, it is imperative to carefully consider international ethical guidelines for biobank activities—such as the *International Ethical Guidelines for Health-related Research Involving Humans* issued by the Council for International Organizations of Medical Sciences (CIOMS, 2016) in collaboration with the World Health Organization (WHO)—particularly in the absence of unified regulations and standards among Latin American biobanks. The 2016 version of these guidelines is recognized for its robust international ethical consensus. They include ethical criteria for the use of biological samples in health-related research involving humans under Guideline 11, titled “Collection, Storage, and Use of Biological Materials and Related Data.” This guideline categorizes biobanks into three types: population biobanks, sample collections for specific projects, and sample collections for clinical, pathological, and forensic purposes. It specifies distinct informed consent options and procedures for sample donors for each type.

In addition, it recommends broad consent for sample collection, as it is best suited for longitudinal studies. However, broad consent is only viable if supported by appropriate governance and responsible biobank management. The guideline provides a detailed set of governance requirements and essential elements that broad consent must include. It discourages specific consent, which, although seemingly ideal for protecting donors, is impractical for future studies. Finally, it also regards general (blanket) consent or open-ended donation as questionable or even unacceptable under ethical standards due to the uncertainty about the future use of samples.

## 5 Discussion

The evolution of biobanks has consistently challenged traditional ethical and regulatory principles in scientific research involving human subjects (50, 51). These challenges largely arise from the nature of biobanks, which facilitate numerous future research projects across broad areas of investigation using highly sensitive samples and information, such as genetic data, that can be shared internationally. The specific purpose of these projects often cannot be known by the donor at the time of donation. The key challenge, therefore, is to strike a balance between the immense social value that biological material and associated data hold for generating significant benefits to human health, enhancing the quality of science, and fostering international collaboration, and the risks posed to each individual donor.

Biobanks must ensure high standards of quality, quantity, and the rapid availability of samples and their associated information to produce the best possible results in biomedical research. They must also adhere to ethical standards and fundamental legal principles to respect the rights of sample donors. The transfer of a sample requires the informed consent of the donor, as it involves a part of their body (considered their property, though not commercially tradable) from which highly sensitive personal information, such as genetic data, can be extracted and potentially linked to personal health information. These are ethically and legally protected under the principles of autonomy, confidentiality, and privacy. Furthermore, the use of human biological material raises ethical and legal considerations related to the patentability and commerciability of research outcomes, as well as the obligation to return research benefits to the community, in line with the principle of distributive justice (51).

In a world facing increasing risks of global pandemics, expanding biobanks capable of interoperating at a global level is a strategic public health priority for LATAM countries. This expansion is contingent upon agreements on fundamental principles such as equitable access to samples, transparency in all processes, adherence to common ethical standards, and respect for local legislation, among other principles of fair collaborative research (52).

We believe that by implementing both the proposals and the guidelines detailed previously, LATAM could gradually integrate itself into the global research ecosystem, not only by improving the interoperability, governance, and coordination of its biobanks but also by adopting minimum validated international standards for their regulation.

As biobanks in LATAM are strengthened and their networked operations are established, high-quality human biological material and associated data will become available for local research and participation in international medical research, facilitating the integration of countries in the region into the global research ecosystem. At the same time, having reference biobanks in various LATAM countries will ensure not only the reproducibility of biomedical and clinical studies but also foster the local development of innovative drugs, vaccines, and diagnostic tests tailored to the region’s health needs. To achieve these goals, it is necessary to agree on common standards for the harmonization and interoperability of the LABN in coordination with each country’s regulatory agencies.

In this context, the challenge for LATAM countries is to incorporate into their respective regulatory agencies—within the regulatory processes for both preclinical and clinical studies—the international technical, ethical, and legal standards for biobanks mentioned earlier. These standards will not only help improve the reproducibility of analytical research data—a prerequisite for data reuse in the context of open science—but also provide LATAM biotechnology companies with the capacity to innovate with *in vitro* diagnostic tests and other biotechnologies that are more accessible to the regional market, while meeting international regulatory requirements, such as the European regulation on *in vitro* diagnostic medical devices [Regulation (EU) 2017/746] (3). Additionally, research centers in the region will be able to participate as active partners in multicenter clinical studies, rather than merely serving as passive providers of samples and associated data. In this sense, biobanks should be recognized as reliable partners in international collaborative research and as essential platforms for the biotechnology industry in our region, enabling autonomous and competitive development in international biotechnology markets.

LATAM has a unique opportunity to develop biobanks due to its geographic, climatic, ethnic, and socio-demographic diversity, which results in varying epidemiological profiles across countries. This diversity is evident in healthy populations, at-risk groups, and those with illnesses. By capturing a detailed, large-scale view of the population’s biology, the region can generate accurate data to inform policies for prevention, early diagnosis, treatment, and rehabilitation.

As LATAM biobanks can work in a coordinated and harmonized way, by providing the scientific community with high-value and high-quality biological resources and data from the LATAM population, a LABN can improve national and international biomedical and clinical research, promote innovation and enhance regional health policies. On the other hand, counting on a biobanking ecosystem will foster effective communication and collaboration among researchers, scientific community, biotechnological companies, and government agencies. This can drive scientific discoveries that critically improve human health conditions both at a regional and global level.

We have identified key regulatory, policy, and infrastructure gaps that LATAM must address to achieve harmonized interoperability standards. Such harmonization, along with the implementation of the proposed measures and adherence to international guidelines, is critical for replicating biomedical studies, establishing minimum regulatory standards for biobanks and medical products globally, and fully integrating LATAM into the global research ecosystem.

## Footnotes

^1^Some of these institutions in LATAM still do not meet ISO standards of biobanking, so they are biorepositories housing human samples for specific research purposes.

^2^ https://www.gov.br/conselho-nacional-de-saude/pt-br/acesso-a-informacao/sobre-o-conselho/camaras-tecnicas-e-comissoes/conep/biobancos

^3^https://www.isber.org/page/BPR (Published in 2023).
